# Condition-Dependent Effects of Mating on Longevity and Fecundity of Female Medflies: The Interplay between Nutrition and Age of Mating

**DOI:** 10.1371/journal.pone.0070181

**Published:** 2013-07-24

**Authors:** Stella A. Papanastasiou, Christos T. Nakas, James R. Carey, Nikos T. Papadopoulos

**Affiliations:** 1 Department of Agriculture, Crop Production and Rural Environment, University of Thessaly, Volos, Magnisia, Greece; 2 Department of Entomology, University of California Davis, Davis, California, United States of America; University of Crete, Greece

## Abstract

**Background:**

In various species mating exerts direct and indirect effects on female demographic traits ranging from life span shortening to behavioural shifts. A wealth of data regarding effects of nutrition on longevity and reproduction output also exists. Nonetheless, little is known regarding the interaction between the age of mating and nutrition on female fitness.

**Methodology:**

We studied, the effects of protein deprivation and age of mating on female fitness traits, using a wild population of the Mediterranean fruit fly (medfly). We tested the hypotheses that (a) protein availability increases female lifespan and fecundity, (b) female longevity and egg production are independent of mating and the age of mating, and (c) female mating behaviour is independent of their age and nutritional status. Thus, we recorded the mating success and the copulation characteristics, as well as the egg production and survival of females mated at young or at old age and fed a full or a protein-deprived diet.

**Results:**

Mating boosts egg production and reduces longevity of protein-fed females. On the contrary, mating increases the longevity of protein-deprived females. Mortality responses (negative or positive) to mating are expressed after a long lag phase. Old females are more receptive and less selective than young females regardless of the food regime.

**Conclusions:**

Our findings suggest that condition (nutritional status and age) defines the positive or negative output of mating in female medflies. These results contribute towards understanding the effects of mating, aging, resource allocation and their interactions on survival and female reproduction.

## Introduction

Mating in insects induces both direct and indirect effects on females ranging from life span reduction to behavioural changes. For example, specific components of male *Drosophila melanogaster* ejaculates increase egg production and reduce female longevity [1], [2]. Likewise, mating and male accessory fluids alter female behaviour such as decreasing mating receptivity [3]. However, in several Diptera species mating does not affect either female survival or egg production ([4] and references therein). Additionally, the age of mating is a major determinant of reproductive success and female fitness [5], [6]. Although the effects of nutrition on female fitness are well known, there are few studies on the interaction between the age of mating and nutrition on female longevity and reproduction.

In the adult stage of insects, nutrients derived from foraging behaviour, and often from larval reserves, are used mainly for reproduction, locomotion, and soma maintenance. The allocation of energy depends on adult activity, age and sex related needs as well as on environmental factors (reviewed in [7]). Resource trade-offs among life history traits are present in both rich and stressful environments. However, the costs deriving from such trade-offs are more pronounced in stressful environments. Therefore, although resource allocation toward soma maintenance or reproduction usually imposes a cost on fecundity or lifespan, *ad libitum* availability of a nutrient-rich diet enhances both fecundity and longevity of females of several insect species [8], [9]. On the other hand, dietary restriction has been reported to extend the lifespan in *Drosophila* species [10] and other organisms [11], [12]. The latter is usually observed during short periods of nutritional deprivation and is caused by a reversible resource allocation to maintenance with a parallel inhibition of reproduction [10], [11], [12].

Diet strongly influences the sexual behaviour of various arthropods [13], [14], [15]. Protein deprivation may weaken female selectivity for mating partner and increase receptivity [15], [16]. Alternatively, protein availability may boost the sexual activity of both sexes and increase female receptivity [17]. Previous behavioural studies on Tephritids’ mating, addressing the effect of diet, mainly focus on the impact of protein deprivation of both sexes on female remating inhibition and/or refractory period and copulation characteristics [17], [18]. Several studies on dietary effects on male sexual behaviour and attractiveness reported that protein-deprived males are discriminated against by potential mating partners as reflected in their higher frequencies of mating rejection, shorter copulations, and more remating attempts [19], [20], [21]. Remarkably, there are no similar studies for female insects concerning the direct and interactive effects of diet and age on female sexual behaviour (i.e. sexual receptivity, copulation duration, latency).

Although age associated changes of male mating success have been addressed in various insect species [18], [22], [23], [24] less attention has been given on effects of age on female sexual and reproductive behaviour [4], [25]. As insects age and senescence prevails the mortality risk increases, fecundity rates decrease [26], and female mating receptivity increases due to progressively limited opportunities of reproduction [27]. Consequently, females tend to become less choosy as they grow older, because the probability to increase their fitness by succeeding copulations decreases [28], [29]. On the other hand, in a variety of insect species where females produce eggs irrespectively of copulation, female tendency to mate may decrease with age due to a “switch” of the mating – sexual behaviour to an egg laying behaviour [30]. In this group of insects, the interactions between female age at mating and nutrition, as well as possible interactions with previous reproductive output remain to be investigated.

Trade-offs between the energetically demanding reproduction (copulation, egg production) and longevity (soma maintenance) have been determined in many organisms including insects belonging to different classes [4], [31], [32], [33]. Virgin or irradiated *D. melanogaster* females live longer than mated or egg producing females, indicating a separate cost of mating and egg production [1], [34]. On the other hand, mating has no negative effect on the lifespan of *Saltella sphondylli* females despite a reported cost of egg production [35]. Age of the first mating that is directly related with the reproductive maturity, defines the organism’s reproductive period, affects the equilibrium between reproduction and longevity, and regulates the fitness output [36], [37].

The Mediterranean fruit fly is a model organism intensively used for demographic research [38], [39], [40]. Dietary restriction has a neutral [41] or a negative effect on demographic traits of female medflies that is reversible when protein is provided even at advanced ages [42]. Female oviposition opportunities determine the magnitude of the negative effects of dietary restriction on medfly longevity [41]. Studies regarding effects of mating on lifespan and egg production result in controversial conclusions. Chapman and co-workers (1998) [4], comparing fecundity rates between mated and virgin females, proposed an independent cost of mating from egg production. Earlier studies suggest an indirect cost of egg production on female longevity without, though, testing effects of mating [43], [44]. Conversely, two additional studies reported no effect of mating on female survival and a positive effect of mating on fecundity [45], [46]. However, in all of these studies females were fed a full diet and, therefore, none of the above examines the interaction between mating and nutritional status.

In the present study, we manipulated two vital conditions for female fitness (nutrition and age of first copulation) to determine effects of mating on female sexual receptivity, lifespan and fecundity. We compared the fitness components of females mated when young (15 days old) or old (40 days old) and fed a protein-rich or a protein-deprived diet. Copulation duration and latency time of young and old females were also recorded. We tested the hypotheses that (a) protein availability increases female lifespan and fecundity, (b) female longevity and egg production are independent of mating and the age of mating, and (c) female mating receptivity, copulation duration and latency time are independent of their age and nutritional status. Therefore, we predicted similar longevities and fecundities between mated, at young or old age, and unmated females within each food regime. We also expected young and old females to mate at similar proportions and to present similar copulation duration and latency time regardless of their diet.

## Materials and Methods

### Ethics Statement

This study was conducted in the laboratory using flies collected from the wild. There is no specific permission required for collecting wild medflies, since this is a major pest of fruit trees in the area and is, therefore, neither an endangered nor a protected species.

### Experimental Conditions and Flies Used

The medflies used in our experiments were obtained from field-infested fruits collected in the area of Volos, Greece. Flies were reared in laboratory conditions (25±1°C, 65±5% R.H., and a photoperiod of L14:D10 with photophase starting at 0700 h.) for 1–3 generations (F_1_– F_3_). Details of the rearing method are given by Diamantidis and co- workers (2008) [47].

Flies were sorted by sex within 24 hours after adult emergence. Males were maintained until the testing dates in 20×20×20 cm Plexiglas cages (maximum 20 individuals per cage) and were provided a full adult diet [yeast hydrolysate (MP Biomedicals LLC., France), sugar (sucrose) and water at 1∶4:5 weight ratio, respectively]. Females were placed in individual cages (400 ml plastic transparent caps) with an artificial oviposition substrate [5 cm diameter hollow, plastic red coloured hemisphere (dome) bearing 40–50 evenly distributed holes (1 mm diameter)]. All flies had free access to water and adult food (specifics of female diet are given below). Additional details regarding individual cages and oviposition devices are given in Sarakatsanou et al. (2011) [48].

### Female Groups

A total of 700 individually stored females was randomly divided in two groups and assigned to different nutritional regimes. Specifically, 320 females were fed a full adult diet [yeast hydrolysate (MP Biomedicals LLC., France), sugar (sucrose), and water at 1∶4:5 weight ratio, respectively], and 380 females were fed only sugar [sugar (sucrose) and water at 1∶3 weight ratio]. Females fed the full diet are hereafter referred as “protein-fed females” while females fed only sugar as “protein-deprived” or “protein-restricted” individuals. Female mortality and egg production were recorded daily.

### Mating Tests

Sixty protein-fed and 90 sugar-fed randomly chosen females were not given any mating opportunity over their entire lifespan (control females). The sexual receptivity of female medflies, fed either a full or a protein-deprived diet, was tested at two distinct ages: 15 days old (young females) and 40 days old (old females). By the age of 15 days female medflies are sexually mature [49] while at the age of 40 days the beginning of mortality increase occurs in wild medflies populations [50]. At 0630 on the test day, 3 non-mated, sexually mature males (12–14 days old) were placed in each individual cage containing a virgin female. Once the copulation had initiated, the two spare males were removed from the cage, and the newly formed couple was allowed to complete the copulation. Observations were taken every 15 minutes from 0700 to 1600. The number of copulations for each treatment, the latency time, and the duration were recorded. In the current study we used a wild population of medfly that usually exhibits an extended latency to mate time. Due to the long presence of males in the individual cages during the mating tests (in some cases approaching 9 hours before being engaged to copulation) a crowding effect could bias our data. For this reason, the effect of crowding conditions in the treatment cages was simulated for the control females by adding 3 marked females in each cage, for as long as the mating tests lasted. After the mating test, 3 subgroups of females were created: mated, not mated, and control.

### Statistical Analyses

Data analyses were performed using SPSS 20.0 (SPSS Inc., Chicago, IL, U.S.A.) and Stata 11.2 (StataCorp, College Station, TX, USA). Differences of female mating receptivity in relation to the type of diet and age were assessed through logistic regression analysis. The effect of female age and diet on mating duration and the latency time were evaluated using the independent samples *t*-test. The effect of food, mating status, and age of mating on the fecundity were tested with the non-parametric Kruskal – Wallis test. The Wilcoxon-Mann-Whitney (WMW) test was used as post-hoc with a Bonferroni adjustment. We applied the Cox proportional hazards model to determine the effect of diet and age of mating on the longevity of females. In order to avoid discretization bias, caused by the aggregation of lifespans, which occurs in lifetime data, smoothed mortality rates were calculated for all treatments along the lines proposed in Muller et al. (1997) [51]. This strategy was used because our dataset has the same quantitative characteristics and properties as the dataset of Muller et al. (1997) [51]. The same smoothing procedure was followed for the assessment of fecundity rates (e.g. Fig. S2).

## Results

### Effect of Diet and Age on Female Mating Receptivity

**Figure 1:**
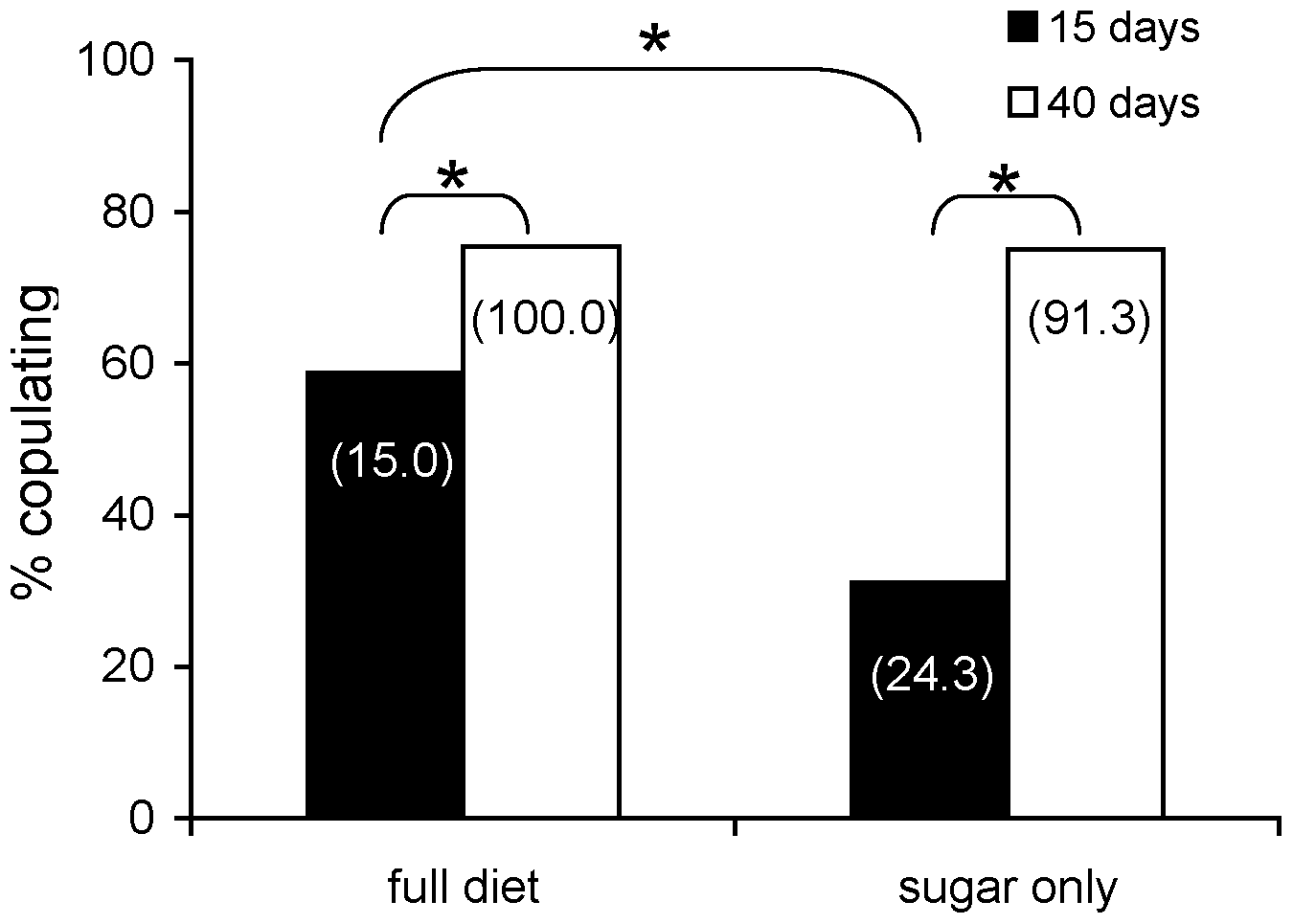
Copulation success of females fed a full (left) and a protein-deprived (right) diet, at a young (15 days) and an old (40 days) age. The percentage of the females that had initiated egg production one day before the mating test is given in parenthesis. Asterisks (*) indicate significant differences (*P*<0.05).

Logistic regression analysis showed that diet regime significantly affected the mating receptivity of females (Wald test *t = *12.212, *P*<0.001). Females fed the full diet were more prone to mate than protein-deprived ones regardless of their age. Differences between diets were more pronounced at 15 days old females (Fig. 1). Age was also a significant predictor of female receptivity (Wald test *t = *35.697, *P*<0.001). The mating propensity of old females (40 days old) was significantly higher than that of young females (15 days old) in both diet regimes (Fig. 1). Moreover, the interaction between the adult diet and age of mating was significant as well (Wald test *t = *6.776, *P*<0.05), indicating that the reduced mating receptivity at young age was significantly higher for protein-deprived females.

### Effect of Diet and Age on Copulation Duration and Latency

**Figure 2:**
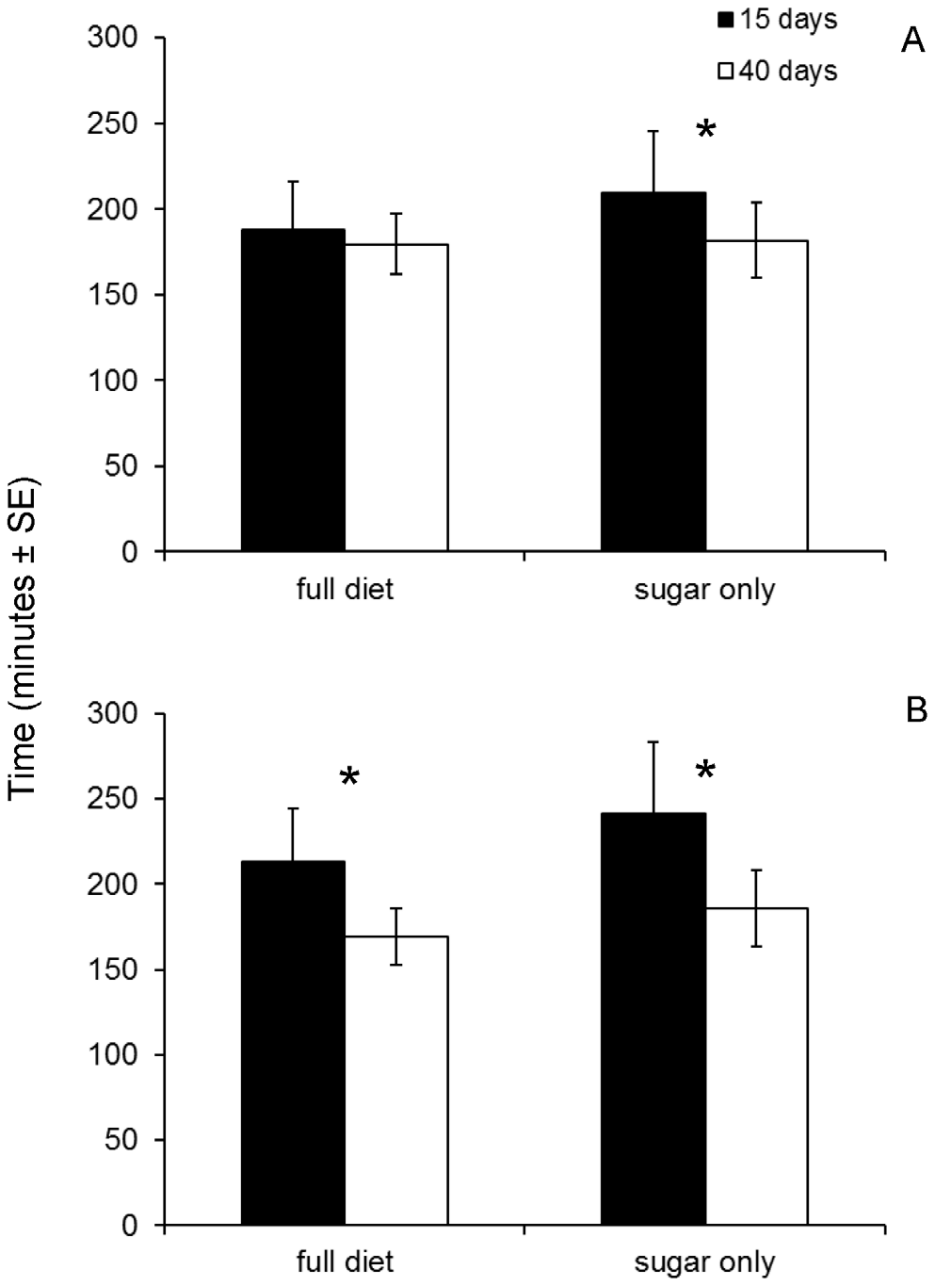
Copulation duration (A) and latency time (B) of females fed a full (left) and a protein-deprived (right) diet. Asterisks (*) indicate significant differences (t-test, *P*<0.05).

Copulation duration was not affected by the nutritional status of females (*P*>0.05) but was significantly longer for the young females (*t* = −2.287, df = 252, *P*<0.05) regardless of the diet regime. However, assessing the effect of age of mating on copulation duration within each diet regime we found a significantly longer copulation only for the young protein-deprived females (*t* = 2.672, df = 100, *P*<0.05) (Fig. 2). Similarly, based on data pooled over the two female age categories, the nutritional status of females did not affect the copulation latency time (*P*>0.05). However, young females were significantly choosier than old females regardless of the diet (*t* = −2.974, df = 252, *P*<0.05) and within each diet regime (*t_protein_* = 2.16, df = 149, *P*<0.05, *t_sugar_* = 2.0, df = 100, *P*<0.05) (Fig. 2).

### Effect of Diet and Mating Status on Longevity

**Figure 3:**
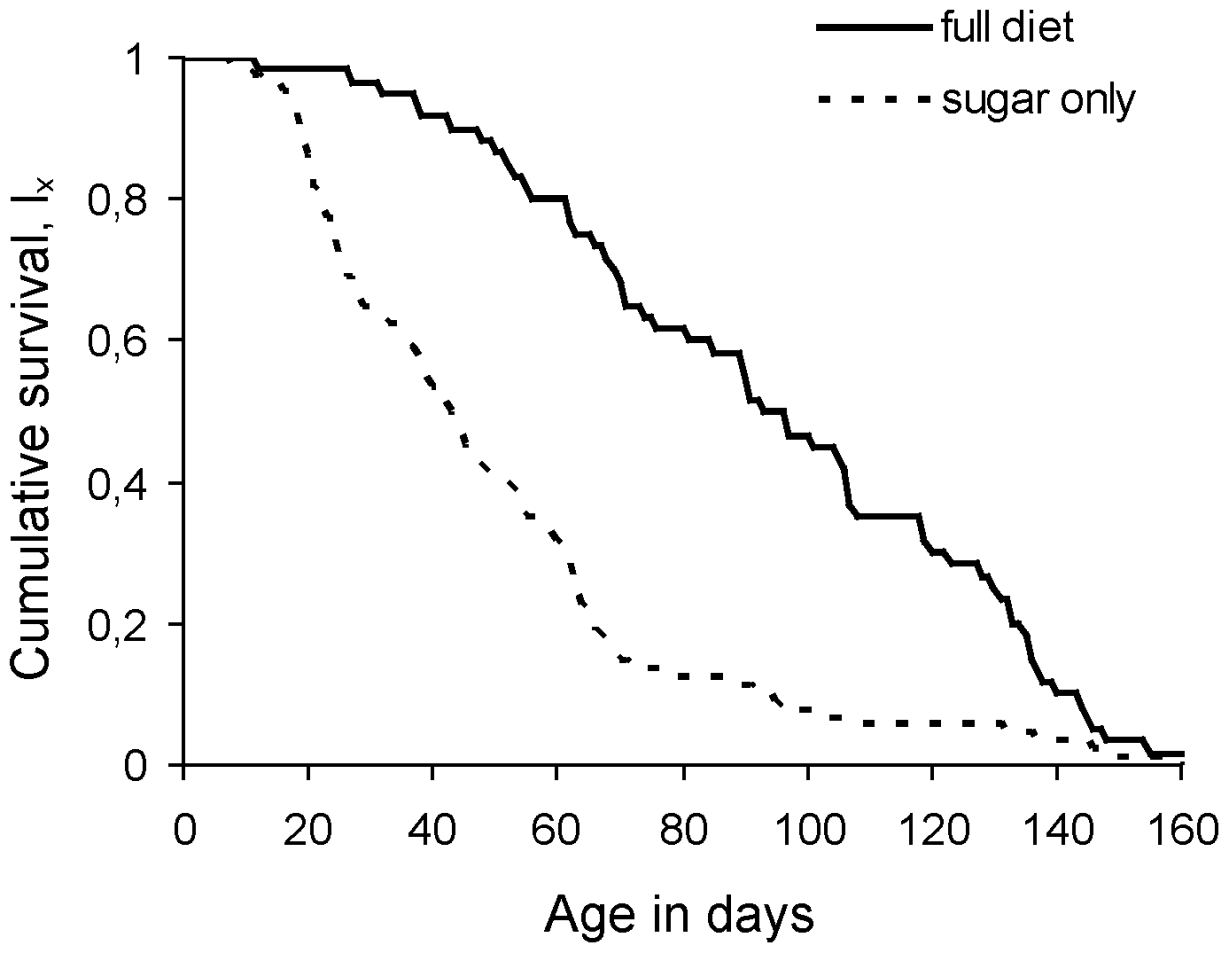
Survivorship of control females fed a full (continuous line) and a protein-deprived (dotted line) diet. Control females were not given the opportunity to mate and were kept virgin throughout lifespan.

Protein availability in adult diet significantly enhanced female survival (considering both control and treatment individuals) regardless of the mating status (Wald test *t* = 230.7, df = 1, *P*<0.001) (Table 1). The results were similar when the effect of food on the survival of control (virgin) females was tested separately (Wald test *t* = 33.7, df = 1, *P*<0.001) (Fig. 3). Mating was also a significant predictor of female lifespan irrespective of the diet and the age of mating (Wald test *t* = 18.5, df = 1, *P*<0.001). Overall, virgin females lived longer than mated ones. Finally, the Cox proportional hazard model revealed a significant interaction between diet and mating status (Wald test *t* = 31.264, df = 1, *P*<0.001) indicating that mating increased mortality rates of protein-fed and decreased mortality rates of protein-deprived females.

**Table 1:** Remaining life expectancy (expected number of days remaining at day x) for females fed either a full or a protein-deprived diet that mated or remained unmated at day 15 or 40.

	Remaining life expectancy (days)						
	0 days	15 days	30 days	40 days	50 days	60 days	70 days
**Full diet**							
mated 40				49.28 (104)	40.11 (102)	32.63 (95)	28.08 (79)
unmated 40				56.41 (34)	46.41 (34)	39.06 (32)	33.82 (28)
mated 15		79.76 (47)	66.46 (46)	59.07 (44)	50.43 (43)	41.43 (42)	33.10 (40)
unmated 15		84.66 (32)	75.00 (30)	67.29 (29)	57.29 (29)	49.25 (28)	40.94 (27)
control	94.78 (60)	81.19 (59)	67.40 (58)	60.77 (55)	53.90 (52)	48.10 (48)	45.43 (41)
**Sugar only**							
mated 40				29.86 (69)	19.86 (69)	12.07 (58)	17.82 (19)
unmated 40				23.46 (23)	14.32 (22)	5.45 (19)	– (2)
mated 15		21.50 (33)	15.61 (19)	12.41 (11)	– (1)	– (1)	– (1)
unmated 15		21.31 (73)	13.58 (48)	7.31 (32)	21.30 (5)	– (2)	– (1)
control	48.49 (90)	35.30 (84)	34.03 (57)	30.16 (47)	29.19 (35)	26.28 (27)	40.58 (13)

Sample sizes of each age class are given in parenthesis.

**Figure 4:**
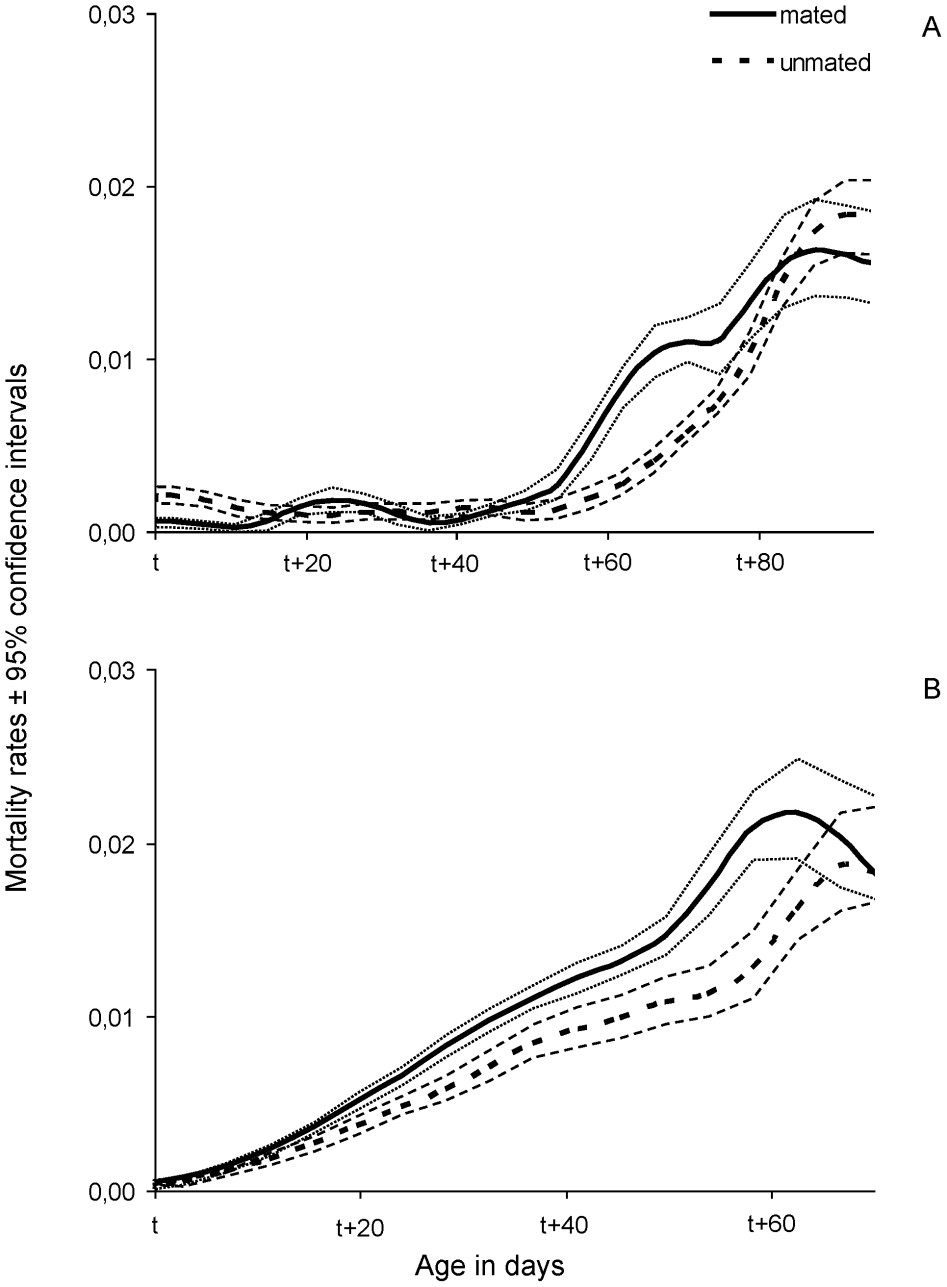
Smoothed mortality rates with 95% confidence intervals of females fed a full diet that mated (continuous line) or remained unmated (dotted line) at t = 15 (A) and t = 40 (B) days.

**Figure 5:**
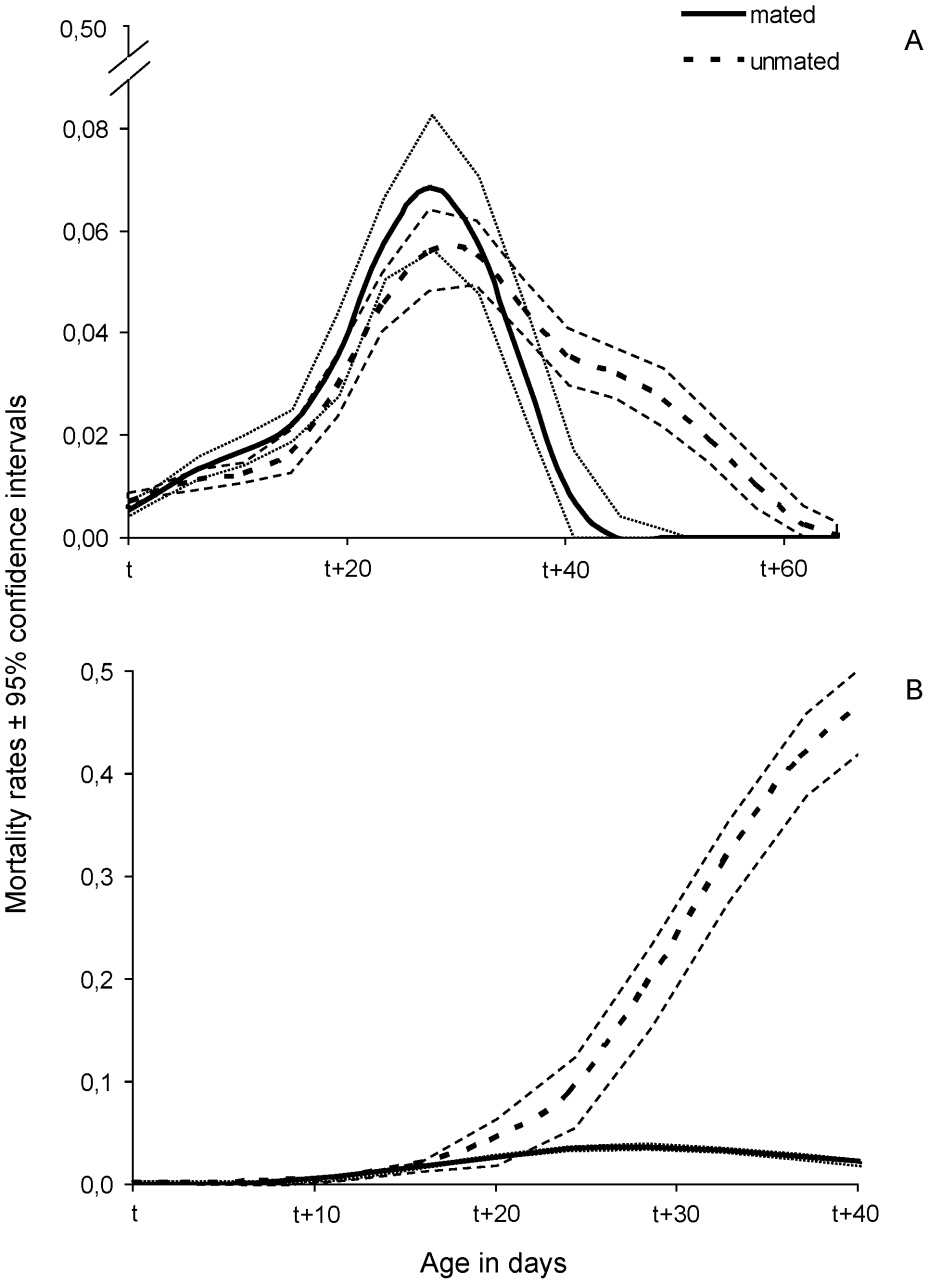
Smoothed mortality rates with 95% confidence intervals of females fed a protein-deprived diet that mated (continuous line) or remained unmated (dotted line) at t = 15 (A) and t = 40 (B) days old.

Smoothed hazard rates were also estimated in order to compare the mortality of mated and unmated females within each age and diet group (Fig. 4, 5) (Table 1). Mated females, fed a full diet, exhibited higher mortality rates than unmated ones, which was more pronounced when mating took place at 40 days old (Fig. 4). In contrast, mated females fed a protein-deprived diet displayed lower mortality rates, which again were more evident for the females mated at older age (Wald test *t* = 3.607, df = 1, *P* = 0.05) (Fig. 5). In both protein-fed and protein-deprived females, a lag phase was observed before differences in mortality rates were expressed, which in all cases coincided with the age of 55–60 days.

Cox proportional hazards model failed to detect differences in post mating longevity between protein-fed females mated at young and old ages. The smoothed mortality rate functions in Fig. S1A suggest that the increase in the hazard rate at older post-mating ages occurs slightly later for the mated at young age protein-fed females. On the other hand, patterns of mortality rates differed significantly in protein-deprived flies that mated at young and old age (Wald test *t* = 51.553, df = 1, *P*<0.001). Age specific mortality rates were low through a 15-day post mating period for flies mated at both ages and picked 25 days post mating. The mortality of females mated at young age was higher than that of females mated at old age through a period of 40 days post mating (Fig. S1B).

### Effect of Diet, Mating Status, and Age on Fecundity

**Figure 6:**
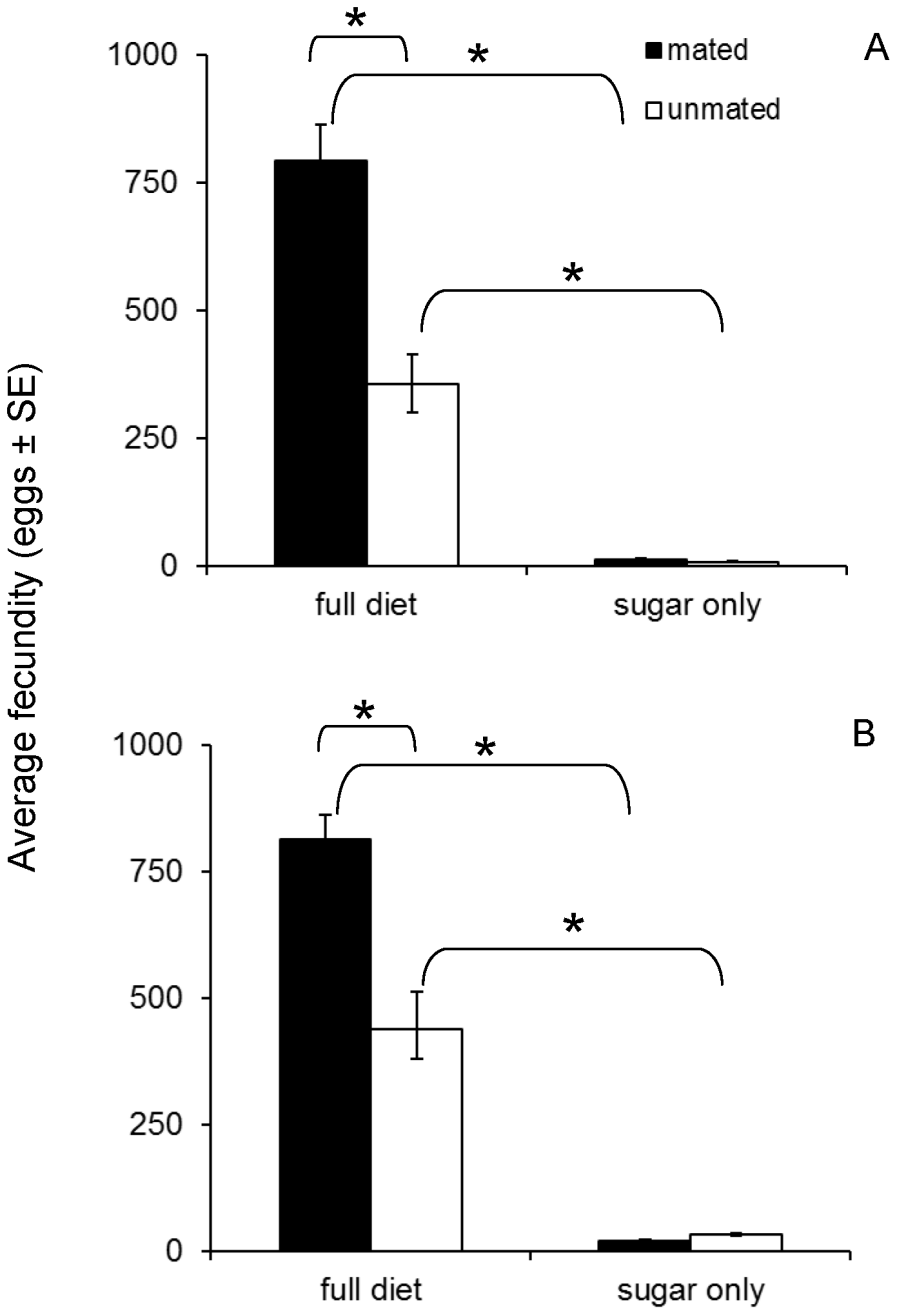
Average number of eggs (±SE) laid by mated and unmated females fed a full or a protein-deprived diet when subjected to mating tests at 15 (A) and 40 (B) days of age. Asterisks (*) indicate significant differences (*P*<0.05).

Females fed a full diet oviposited significantly more eggs than protein-deprived females regardless of the mating status and age of mating (Kruskal-Wallis test, *P*<0.001) (Fig. 6). Pair wise comparisons within each age group indicated that among protein-fed females, mated females exhibited a significantly higher fecundity than unmated ones (WMW test, *P*<0.001) (Fig. 6, Table 2). Either mated or virgin protein-fed females oviposited more eggs than alike females fed only sugar (WMW test, *P*<0.001 for both comparisons). Post mating fecundity of protein-fed females that were mated either at 15 or at 40 days was significantly higher than that of females that remained unmated at the same age (WMW test, *P*<0.001).

**Table 2:** Remaining fecundity (expected number of eggs remaining at day x) for females fed either a full or a protein-deprived diet that mated or remained unmated at day 15 or 40.

	Remaining fecundity (eggs per female)						
	0 days	15 days	30 days	40 days	50 days	60 days	70 days
**Full diet**							
mated 40				605.77 (104)	448.38 (102)	309.45 (95)	188.35 (79)
unmated 40				308.59 (34)	244.82 (34)	181.35 (32)	122.18 (28)
mated 15		748.66 (47)	652.53 (46)	515.60 (44)	399.98 (43)	279.28 (42)	157.45 (40)
unmated 15		327.09 (32)	285.36 (30)	248.73 (29)	212.18 (29)	168.39 (28)	126.61 (27)
control	555.03 (60)	548.73 (59)	485.82 (58)	385.65 (55)	293.07 (52)	221.58 (48)	142.57 (41)
**Sugar only**							
mated 40				4.65 (69)	1.10 (69)	0.24 (58)	0.04 (19)
unmated 40				5.52 (23)	1.17 (22)	0.13 (19)	– (2)
mated 15		10.51 (33)	1.06 (19)	0.21 (11)	– (1)	– (1)	– (1)
unmated 15		10.03 (73)	2.12 (48)	0.53 (32)	0.01 (5)	– (2)	– (1)
control	9.84 (90)	8.11 (84)	2.45 (57)	1.30 (47)	0.17 (35)	0.04 (27)	0.00 (13)

Sample sizes of each age class are given in parenthesis.

The fecundity of protein-deprived females that were mated or remained unmated at 15 days old was similar both before and after the mating test and throughout life (*P*>0.05). The results were similar for the protein-deprived females that were mated and remained unmated at 40 days old (Fig. 6). The number of eggs laid by protein-deprived females at both mating ages was extremely low.

**Figure 7:**
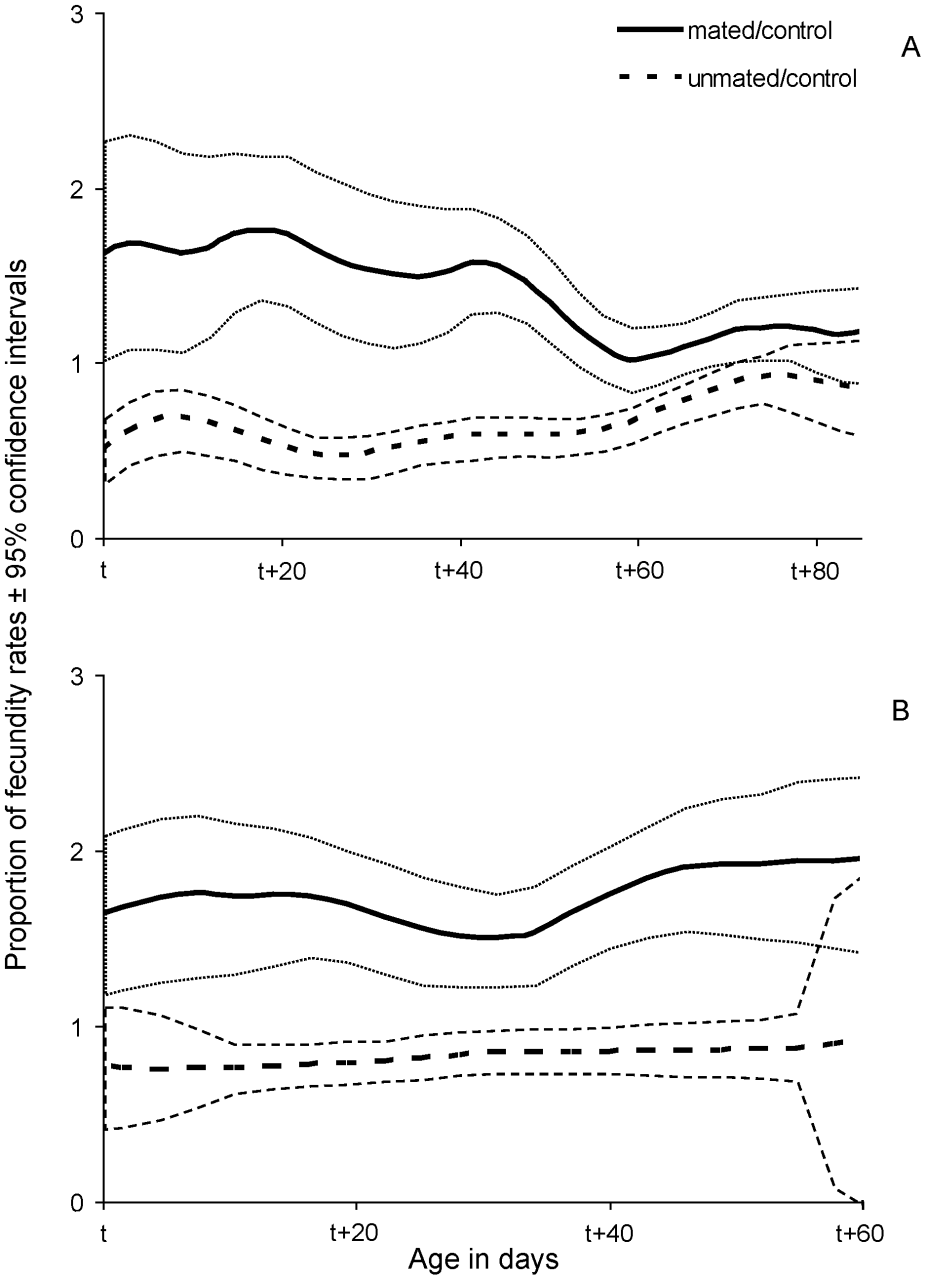
Smoothed proportion of fecundity rates (age specific fecundity of treatment/age specific fecundity of control) with 95% confidence intervals of females fed a full diet that mated (continuous line) or remained unmated (dotted line) at t = 15 (A) and t = 40 (B) days.

**Figure 8:**
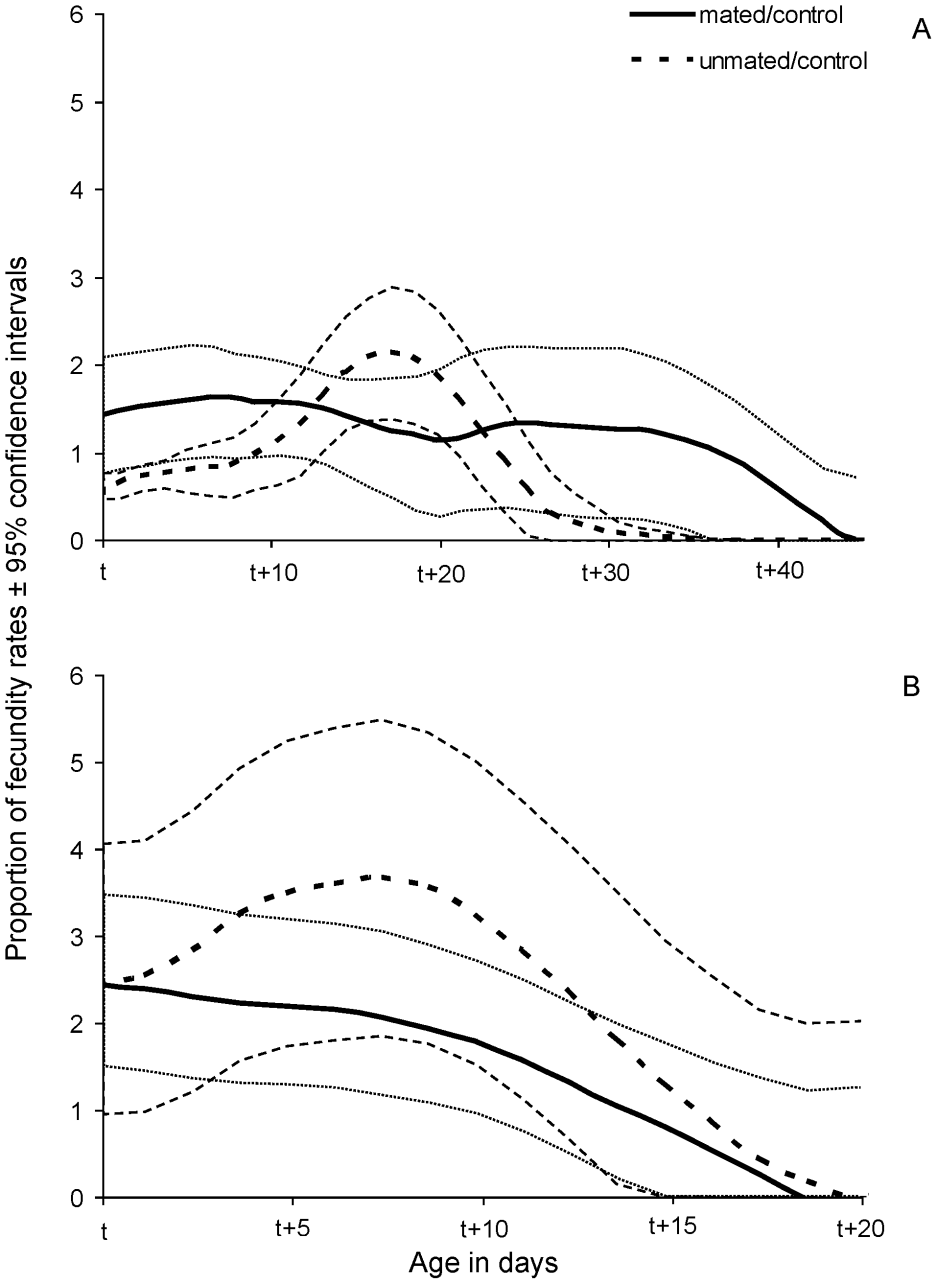
Smoothed proportion of fecundity rates (age specific fecundity of treatment/age specific fecundity of control) with 95% confidence intervals of females fed a protein-deprived diet that mated (continuous line) or remained unmated (dotted line) at t = 15 (A) and t = 40 (B) days old.

In order to assess the effect of mating on the egg production (m_x_) we estimated the proportion of smoothed fecundity rates of mated (m_x, mated_/m_x, control_) and unmated (m_x, unmated_/m_x, control_) females in relation to control females (Fig. 7, 8). Mated, protein-fed females demonstrated stable and significantly higher fecundity rates than unmated ones regardless of the age of mating. The fecundity rates of unmated females were constantly lower than that of control females (Fig. 7). Effects of mating on age specific fecundity rates were not clear for protein-deprived females (Fig. 8). Age specific patterns of egg production are given in Fig. S2.

## Discussion

The results of this study demonstrate that (a) female mating receptivity increases with age and nutritional status, (b) diet does not affect the copulation duration or latency but older females exhibit shorter copulations and are less choosy in mate selection, (c) protein-fed females live longer and lay significantly more eggs than protein-deprived females, (d) mating boosts the egg production of protein-fed females with a subsequent cost in survival, and (e) mating increases the longevity of protein-deprived females, suggesting a protective effect, especially when it takes place in older ages.

### Aging and Protein Availability Increase Mating Receptivity

Aging may modify female sexual behaviour by increasing mating receptivity and decreasing selectivity in several animal and insect species [28], [29], [52], including laboratory adapted, short lived medfly strains [25]. Our data prove that old female medflies (40 days old) are less choosy, and as a consequence their mating propensity is almost twice as high compared to young ones. This may be the result of either the reduction of their discriminatory capacity or, alternatively, females may still be able to evaluate their potential mating partners but they broaden the limits of acceptable male characteristics due to senescence. Since both female life expectancy and residual reproductive value diminish with age, old females readily accept a mating partner in order to ensure reproductive success. On the contrary, a false evaluation of a mating partner by young sexually mature females could relatively easy be compensated by remating with a higher quality male in future attempts.

Adult diet influences the maturation of ovaries and egg production of several female Tephritids [14], [17], [53], and may also regulate female receptivity. Protein availability is also known to increase the mating success of male medflies [21], [54]. Lack of protein reduces the sexual receptivity and attractiveness of female medflies, which are courted by males significantly less than protein-fed females [55]. Our results are consistent with previous findings regarding the relation of adult diet and sexual behaviour and suggest that the mating receptivity of female medflies is positively affected by protein availability in the diet.

Similar studies regarding the effect of the nutritional status on the reproductive behaviour of rodents have led to two contrasting hypotheses. According to the “metabolic fuels” hypothesis, the animal sacrifices reproduction in periods of scarce food availability. On the other hand, animals that fulfil the “reproduction at any cost” hypothesis will not sacrifice reproduction even when sufficient food is unavailable [12]. Energetic investment in offspring production differs considerably between the two sexes. Thus, males may fit the “reproduction at any cost” hypothesis and females of even the same species the “metabolic fuels” hypothesis.

In the present study, protein-deprived female medflies seem to follow the “metabolic fuels” hypothesis when they are young, but their readiness to mate is more similar to the “reproduction at any cost” hypothesis when older. Young protein-deprived females might have been reproductively immature at the age of 15 days exhibiting low mating receptivity; however, our results show that 15 days old, virgin, protein-deprived females have reached reproductive maturity before participating in the mating tests as they had initiated oviposition at higher rates (24.3%) than protein-fed females (15%) (Fig. 1). Earlier studies have shown that, by the age of 14 days, all female medflies fed a full diet are reproductively mature and inseminated [49]. A poor nutritional environment seems to negatively affect female medfly decision to copulate when young, which is later reversed due to aging.

### Female Age but not Diet Affects the Copulation Duration and Latency

Shorter copulations can be advantageous by contributing to lower risk of predation and direct engagement in oviposition [18]. On the other hand, longer copulations may result in an elevated load of sperm transfer. Consequently, both short and long durations in mating could support an increase of fitness. Copula characteristics of several fruit fly species including medfly are strongly affected by adult diet and other conditions [18], [53], [56]. In *Anastrepha* spp., male diet determines the copulation duration and latency time, and protein-fed females participate in shorter copulations. Protein deprivation of laboratory adapted female medflies increases mating duration, indicating a nutritional advantage for females [56]. Nevertheless, protein availability in male medflies diet does not seem to affect copulation duration [54].

Our results reveal no significant effect of diet on copulation duration or latency, which may indicate that female nutritional status is not as important as male nutritional status in affecting mating. However, female age had a high impact on both latency time and duration. Young females delay initiating copulations compared to old females, indicating higher selectivity for mating partner. Also, old females engage in shorter copulations. This behaviour is probably related to their readiness to start ovipositing fertile eggs in an attempt to compensate for the low residual reproductive value due to senescence. Furthermore, male medflies are capable of discriminating among their potential mates based on female age [57]. Thus, old females engaging in shorter copulations may reveal poor evaluation by their male partners, which instead prefer to terminate mating earlier and search for higher quality females (younger). These hypotheses respectively depend on the availability of host fruit for females to oviposit, as well as on the age distribution of the wild population depending on the season [58] and need to be tested in future studies.

### Protein Availability Increases Female Fitness

Previous studies have shown that protein deprivation during immature and/or adult stages affects several fitness traits of medfly, such as adult size, duration of immature stages, successful adult emergence, longevity, and fecundity [55], [59]. It is also known that female fitness increases when a full diet is provided during both immature and adult stages [8], [60], [61]. Our results are in agreement with previous information, since protein-deprived females had a shorter lifespan and laid significantly less eggs than non-deprived females. Similar results were observed when virgin females (control, protein-fed vs protein-deprived) were compared and when protein-deprived females were tested against protein-fed ones regardless of their mating status.

### Mating Triggers Egg Production and Induces Trade-offs Between Fecundity and Longevity only in Protein-fed Females

Mating of both young (15 days old) and old (40 days old) protein-fed females doubled their remaining fecundity rates compared to the unmated females and induced a cost in longevity. Although previous studies draw controversial conclusions regarding the effect of mating on medfly egg production [4], [62], our data provide solid evidences of a surge in egg laying caused by mating which, in addition, increases mortality rates most probably due to a resource allocation to egg production. Contrary to our findings, a negative effect of mating on longevity of female medflies has not been attributed to increased egg production [4]. A similar cost of mating in *D. melanogaster* females has been attributed to male seminal fluids [1]. The fact that the sheer increase in egg laying is independent of females’ age and oviposition history in medfly is intriguing, but whether molecules in male ejaculates are responsible remains to be investigated. Mating-induced increase in egg production of *D. melanogaster* is caused by two peptides contained in male seminal fluids [2]. In *D. pseudoobscura* multiple mating, which may increase the level of sperm load or provide a nutritional advantage, triggers higher fecundity rates [63]. Likewise multiple matings increase fecundity in several tephritid species [46], [64]. The acquisition of the factors responsible for the triggering of egg production after mating, and the indirect cost on longevity in medfly requires further studies on both quantitative and qualitative aspects of sperm and seminal fluids transferred, as well as on their chemical composition.

### Mating Increases the Longevity of Protein-deprived Females

Through mating, apart from sperm transfer, several compounds of the seminal fluids are also transferred to female reproductive tract and may affect egg production and other traits. Nevertheless, the composition of male ejaculates may, in some cases, have a negative impact on the fitness of female mating partners [3]. Proteins contained in the ejaculate secretions have been proven to alter female physiology and behaviour in *D. melanogaster* by suppressing the mating receptivity, reducing lifespan, and mating success. Most importantly a sex peptide of male ejaculate accessory gland proteins (*Acps*) was found to increase mortality and thus decrease fitness of female *D. melanogaster* [1], [3]. However, female negative response to this sex peptide was found to vary in different nutritional environments. Female mortality was more pronounced in a protein-deprived diet, while protein-fed females lived even longer when mated [65]. Therefore, in a broader perspective, mating induces costs or benefits that vary depending on conditions.

Previous experiments on medfly, testing a mass reared strain, indicated a clear cost of mating translated in reduced lifespan [4]. In the same study, when a wild-type stock of insects was tested, no significant differences in survival between virgin and mated females were observed [4]. Our results not only are in contrast to this finding but also clearly show a benefit of mating in protein-deprived females. Apparently, mating may provide a nutritional advantage in malnourished females, through seminal fluids and extend their lifespan. Effects of mating seem to be more dramatic in older individuals. This finding is in agreement with the nutritional environment dependence of mating costs – hypothesis [65]. Effects of mating can be beneficial or deleterious depending on a variety of factors such as diet, condition, age, strain, and their interactions.

Depending on female condition (diet, age) mating can either increase or decrease life span. However, both negative and positive effects are expressed after a lag phase and are more immediate when mating takes place at an older age. Latent, mating-induced costs or benefits occur approximately 40 and 20 days post mating for young and old females, respectively. Young females are more robust and refractory to the costly or beneficial effects of mating. However, frail old individuals are extremely demonstrative in terms of benefits deriving from mating. Concerning the well-nourished females, there might be a “trade-offs” accumulation because of mating-increased egg production that becomes apparent at old ages.

In conclusion, our data demonstrate that effects of mating on vital demographic traits of females such as longevity and fecundity may range from beneficial to harmful depending on female condition (nutritional status and age of mating). This dynamic association between mating and female fitness components raises several proximate and ultimate questions that need to be addressed in future studies.

## Supporting Information

Figure S1 Smoothed mortality rates with 95% confidence intervals of protein-fed (A) and protein-deprived (B) females that mated at t = 15 days (continuous line) or at t = 40 days (dotted line).
(TIFF)

Figure S2 Raw and smoothed (running average with 5-day period) age specific egg production of protein-fed (A) and protein-deprived (B) females that mated or remained unmated at 15 days (first row), at 40 days (second row) and of control females (third row).
(TIFF)
